# *Plesiomonas shigelloides* Septic Shock Leading to Death of Postsplenectomy Patient with Pyruvate Kinase Deficiency and Hemochromatosis

**DOI:** 10.1155/2016/1538501

**Published:** 2016-08-17

**Authors:** Mohammed Samannodi, Andrew Zhao, Yaser Nemshah, Kevin Shiley

**Affiliations:** Department of Medicine, Buffalo Mercy Hospital, University at Buffalo, Buffalo, NY 14220, USA

## Abstract

Although *Plesiomonas shigelloides*, a water-borne bacterium of the Enterobacteriaceae family, usually causes self-limiting gastroenteritis with diarrhea, several cases of sepsis have been reported. We report the case of a 43-year-old male patient with hemochromatosis, pyruvate kinase deficiency, and asplenia via splenectomy who developed septic shock caused by* P*.* shigelloides* complicated by respiratory failure, renal failure, liver failure, and disseminated intravascular coagulation. Early aggressive antimicrobial therapy and resuscitation measures were unsuccessful and the patient passed away. We kindly suggest clinicians to implement early diagnosis of septic shock, empirical coverage with antibiotics, and prompt volume resuscitation based on the high mortality rate of *P. shigelloides* bacteremia.

## 1. Introduction

*Plesiomonas shigelloides* is an oxidase-positive, anaerobic gram negative bacillus bacteria of the Enterobacteriaceae family which normally reside in soil and fresh water environments including the Great Lakes.* P*.* shigelloides* is also distributed among warm- and cold-blooded animals like dogs and seafood. Its method of infection is contamination of the gastrointestinal tract via consumption of raw fish or water-contaminated foods [1–6].

*Plesiomonas shigelloides* usually causes self-limited diarrheal illness but can also lead to extraintestinal infections with immunocompromised patients and patients with underlying hepatobiliary disease like hemochromatosis [7–13]. Bacteremia caused by* P*.* shigelloides* is very rare with only 36 reported cases with our case being the 36th. In addition, the literature has shown a significant mortality associated with* P*.* shigelloides* sepsis in 16 cases with ours being the latest one [7, 8, 10, 11, 13–16].

## 2. Case Presentation

A 43-year-old Caucasian gentleman with past medical history of homozygous hereditary hemochromatosis and pyruvate kinase deficiency leading to a splenectomy at 4 years of age was admitted to the Buffalo Mercy Hospital with a three-hour history of fever, chills, and generalized weakness. The patient denied any recent travel or sick contacts. The only recent change at home was the addition of a new puppy that infrequently bit and scratched him. The patient also reported that one week ago he had eaten home-cooked clams. Lastly, he swam in Lake Erie on many occasions over the past month. During that period of time, local health officials had closed off that particular beach several times because of elevated coliform levels.

On examination, he had a temperature of 39.5°C, heart rate 120 beats/min, blood pressure 80/50 mmHg, and respiratory rate 18 breaths/min. The rest of his dental, chest, abdomen, skin, and neurological exams including meningeal signs were unremarkable. A complete blood count revealed leukocytosis with bandemia (WBC 17.1, bands 28%) and macrocytic anemia (Hgb 8.3 g/dL, MCV: 129.5 dL) while a complete metabolic panel showed renal failure (BUN 18 mg/dL, creatinine 3.55 mg/dL) and liver failure (ALT 2500 U/I, AST 5000 U/I, total bilirubin 14.5 mg/dL, indirect bilirubin 10 mg/dL, and ALP 262 U/I). Additional laboratory findings included lactic acidosis (179.1 mg/dL) with ABG showing high anion gap metabolic acidosis. Electrocardiogram showed only sinus tachycardia. Transthoracic echocardiogram was unremarkable. Computed tomograms of head, chest, and abdomen were unremarkable. Two blood cultures and urine culture were performed. The patient was diagnosed with septic shock.

Aggressive volume resuscitation measures were started along with empiric coverage utilizing intravenous ceftriaxone and vancomycin. The patient developed respiratory failure soon after. Because of worsening renal failure, he also later underwent hemodialysis. On the second day of admission, the blood cultures returned and showed gram negative rods while the urine culture was negative. Total iron and ferritin were performed and returned 196 *μ*g/dL and 1350.2 mg/mL respectively. Disseminated intravascular coagulopathy developed (PTT 74.2 seconds, PT 37.1 seconds, INR 3.2 U/I, fibrinogen 40 mg/dL, fibrinogen degradation products 60 *μ*g/mL, and platelets 55) and the multiorgan failure continued to worsen. As the patient's condition continued to deteriorate, levofloxacin and tobramycin were added to the ongoing antibiotic therapy. Using VITEK2 for microbial identification and antibiotic susceptibility testing, the final blood culture identified the gram negative rods as* Plesiomonas shigelloides*. The organism was found to be susceptible to a multitude of different antibiotics including the patient's regimen of ceftriaxone, levofloxacin, and tobramycin (Table 1). Despite aggressive attempts at resuscitation, the patient's condition further worsened and he passed away on the third day of hospitalization.

## 3. Discussion

In this case report, the patient had three possible sources for* P*.* shigelloides* infection: the addition of a new puppy, recent consumption of clams, and illegal swimming in Lake Erie. In addition, he also had a medical history of homozygous hereditary hemochromatosis and pyruvate kinase deficiency which led to a splenectomy during childhood. The latter condition played a role by increasing the patient's risk of infection while compromising his immune response to bacteria. The former pointed towards previous cases that also involved patients with haematological conditions, including thalassemia intermedia, hemochromatosis, and sickle beta-zero thalassemia [10, 13, 15]. Another study demonstrated that* P*.* shigelloides* uses heme as a source of iron and it had been hypothesized that chronic iron overload in said conditions can predispose patients to infections [13, 15, 17]. We recommend further studies to be performed in the future particularly to establish the association between* P*.* shigelloides'* heme use system and the effect of chronic iron overload relating to infections.

Bacteremia caused by* Plesiomonas shigelloides* is a very rare complication with 36 reported cases worldwide including this presented case [7, 8, 10, 11, 13–16]. However, out of those 36 cases, 16 patients expired, which demonstrates a mortality rate of 44%. The majority of cases of* P*.* shigelloides* sepsis involve neonates and patients with immunocompromising conditions such as leukemia, immunosuppression after allogeneic bone marrow transplantation, and HIV [7–16]. Based on the nature of these patients and the high mortality rate associated with* P*.* shigelloides* bacteremia, we highly recommend early diagnosis of septic shock and early administration of antimicrobials and volume resuscitation. While our patient did not survive even with multiple antibiotic therapy and resuscitation methods, the risks of bacteremia and septic shock still merit a very similar aggressive approach to treatment.

Finally, although this was not done at the time, we suggest that systemic corticosteroid administration may also be beneficial in cases of refractory shock. Septicemia, especially in association with septic shock, has been known to cause adrenal infraction leading to adrenal insufficiency [18].

## Figures and Tables

**Table 1:** Minimal inhibitory concentrations of various antibiotics for *Plesiomonas shigelloides*.

Antibiotics	Minimal inhibitory concentrations (mg/mL)
Cefepime	≤1
Ceftriaxone	≤1
Ciprofloxacin	≤0.25
Gentamicin	4
Imipenem	≤0.25
Levofloxacin	≤0.12
Piperacillin/tazobac	≤4
Tobramycin	2
Trimethoprim/sulfa	≤20
